# The history of substance abuse disorder in critically ill septic adolescent patients is associated with increased utilization of critical care resources and organ dysfunction

**DOI:** 10.1186/s13613-025-01554-9

**Published:** 2025-10-06

**Authors:** Ning Zhuo

**Affiliations:** Department of Nephrology, The Affiliated Suzhou Hospital of Nanjing Medical University, Suzhou Municipal Hospital, Suzhou, 215002 China

Dear Editor,

We read with interest the recent publication by Markus and colleagues on the impact of substance use disorders on critical care management and health outcomes in septic adolescents [1]. The authors made a valuable contribution to the field, however, there are still some issues in this study that deserve further discussion and clarification.

The evolution of sepsis definition and the inconsistency with coding practices may be a neglected confounding factor. The data in this study span from 2008 to 2023, during which significant changes occurred in sepsis diagnostic criteria [1]. The Sepsis-3 definition released in 2016 radically changed clinical practice: it abandoned the previous standard based on the Systemic Inflammatory Response Syndrome (SIRS), which included non-specific indicators such as fever and tachycardia, and instead emphasized organ dysfunction (total SOFA score of 2 points or more to represent organ dysfunction) [2]. This shift had a profound impact on ICD-10 coding practices: for early cases (2008–2015), coding may have been based on the SIRS criteria [3]. For more recent cases (2016–2023), coding increasingly relied on indicators of organ dysfunction, such as elevated creatinine and hypoxemia. Consequently, cases from 2008 to 2015 may include a large number of “sepsis” cases defined according to the old standards, while cases from 2016 onward are more likely to align with the modern definition. This definitional drift leads to heterogeneity in longitudinal data, making it difficult to compare the disease severity between early and recent cases. If substance abuse disorder (SUD) patients, due to worse baseline health, are more likely to be identified as sepsis under the Sepsis-3 criteria, it could artificially amplify the association between SUD and organ dysfunction.

Secondly, the social and legal changes in the diagnostic criteria for SUD, along with recording bias, may be neglected confounding factors. During the study period (2008–2023), many states in the U.S. gradually legalized cannabis (with recreational cannabis use legalized in Washington and Colorado in 2012), which had two effects on SUD diagnosis records [4]. In legalized areas, physicians may be more forthcoming in documenting Cannabis Use Disorder due to reduced legal risks, while in illegal states, physicians may avoid explicit coding and instead use vague terms (such as “substance use”). As public understanding of SUD progressed (e.g., viewing addiction as a disease rather than a moral failing), patients were more likely to voluntarily disclose their drug use history, and doctors became more proactive in screening. For instance, after 2020, electronic health record systems may have embedded standardized SUD screening tools (such as the CRAFFT questionnaire), which could superficially increase the SUD diagnosis rate, although this may not reflect the true epidemiological trend [5]. Therefore, the “increase” in SUD prevalence in the study may be partially attributed to improved diagnostic practices, rather than an actual rise in drug use. If SUD diagnoses have become more comprehensive in recent years, while early cases had a higher rate of missed diagnoses, the association between SUD and severe resource utilization may have been underestimated (as early SUD patients were not included in the exposed group).

We recommend conducting subgroup analyses by time periods, stratified by sepsis definition (pre- and post-Sepsis-3) and the legal environment for SUD (pre- and post-cannabis legalization), to examine the consistency of the associations. Further sensitivity analyses: reanalyze the data using only post-2016 data to assess the robustness of the results in the Sepsis-3 era. In conclusion, before these issues are clarified, this study’s findings should be interpreted cautiously.

## Data Availability

Not applicable.
